# The Mechanism of Word Satiation in Tibetan Reading: Evidence from Eye Movements

**DOI:** 10.16910/jemr.15.5.3

**Published:** 2022-11-01

**Authors:** Xuling Li, Man Zeng, Lei Gao, Shan Li, Zibei Niu, Danhui Wang, Tianzhi Li, Xuejun Bai, Xiaolei Gao

**Affiliations:** Tibet University, Lhasa, China; Tianjin Normal University, Tianjin, China

**Keywords:** Tibetan reading, word satiation, eye tracking, semantic satiation

## Abstract

Two eye-tracking experiments were used to investigate the mechanism of word satiation in Tibetan reading. The results revealed that, at a low repetition level, gaze duration and total fixation duration in the semantically unrelated condition were significantly longer than in the semantically related condition; at a medium repetition level, reaction time in the semantically related condition was significantly longer than in the semantically unrelated condition; at a high repetition level, the total fixation duration and reaction time in the semantically related condition were significantly longer than in the semantically unrelated condition. However, fixation duration and reaction time showed no significant difference between the similar and dissimilar orthography at any repetition level. These findings imply that there are semantic priming effects in Tibetan reading at a low repetition level, but semantic satiation effects at greater repetition levels, which occur in the late stage of lexical processing.

## Introduction

It is common in everyday life for “familiar words to become strange”, a phenomenon termed word satiation. This means that if one stares at a particular word for an extended period, one may begin to feel strange and eventually lose recognition of it (10). How does word satiation emerge and what is its mechanism? Researchers mainly hold two different views on this, namely the semantic satiation hypothesis and the perceptual satiation hypothesis. The former proposes that satiation effects result from the loss of words’ semantics after readers place constant attention on them (38), while the latter proposes that satiation effects are caused by the loss of words’ morphology after readers’ prolonged visual inspection (6).

In category matching tasks, semantic satiation has been identified in English (3; 38; 39), but not in Chinese (54). Additionally, in lexical decision tasks, orthographic satiation has been observed in Chinese (6; 7), but not in English (28). These examples demonstrate that even when performing the same task in multiple languages, the results are inconsistent. As an alphabetic script, English is composed of letters and written linearly. Its phonology will be activated by orthography directly, then the orthography and phonology access the semantics simultaneously. In turn, Chinese is a type of logographic writing that is stereoscopic and non-linear in spatial arrangement (25). It relies on an orthography-semantics path and requires the “gestalt organization” of orthography (20). Because of the differences in writing styles between English and Chinese, English word satiation is likely to be semantic, whereas Chinese word satiation is likely to be orthographic at the perceptual level. Tibetan is a member of the SinoTibetan language family, as well as the alphabetic writing system. Tibetan consists of four vowels, thirty consonants, and five reverse consonants. These letters combine to create syllables, the fundamental unit of Tibetan writing. Tibetan syllable’s structure is similar to that of the Chinese language. It is written around a “base consonant letter” appended before and after and written up and down, displaying a certain stereoscopic quality. For example, the Tibetan syllable “བ"ོགས་” (tie) consists of six consonants and one vowel. Among them, “◌ོ” is the superscribed vowel, “ག” is the base consonant letter, “བ” is the prefix consonant letter, “ས” is the superscribed consonant letter, “◌ྲ” is the subjoined consonant letter, “ག” is the suffix consonant letter, and “ས” is the post suffix consonant letter. The tsheg, “་”, acts as a separator between syllables. Tibetan written structure also shows the features of from left to right linear development, which is similar to English (48; 13). An illustration from Tibetan is the sentence, “)་*ོད་ཐག་པས་བ"ོགས་ནས་བ/ང་།” (The horse’s limbs were tied by ropes). As such, Tibetan has common elements with both English and Chinese. To the best of our knowledge, no research on the process of word satiation in Tibetan reading has been conducted yet.

Semantic satiation was hypothesized by Lambert (22) using the semantic differential scale; however, it was not found by Neely (28) using the lexical decision task. Additionally, Esposito (9) discovered that there was perceptual satiation in a tachistoscopic search task.

Following these studies, researchers investigated the satiation phenomenon in the category matching task. After 30 repetitions of the priming word, the reaction time of participants became significantly longer, indicating that semantic satiation had occurred (38; 39). Semantic satiation, on the other hand, was not found in the improved category matching task (the manner of repetition changed from visual flashing with verbal repetition to verbal repetition or auditory repetition) (12; 32). Furthermore, in a lexical decision task involving native Mandarin speakers reading Chinese, orthographic satiation was identified at the perceptual level (6; 7). In contrast, in the category matching exam, English-Chinese bilinguals who read Chinese reported semantic satiation (53). In summary, whether reading English or Chinese, satiation effects differ depending on the task. This means that various tasks in one language result in distinct satiation effects.

The above-mentioned tasks belong to the paradigms of behavioral experimental methods. These methods are offline (or non-real-time) measures that conceal cognitive processing details of language (47). Therefore, using them makes it difficult not only to control irrelevant variables such as distraction (27), but also to adapt the high-speed integrated characteristics of language processing because they consider accuracy and reaction time as dependent variables (49). By comparison, eye-tracking technology belongs to online (or real-time) measures (35). Hence, not only can it control irrelevant variables such as distraction, but also probe the underlying language cognitive processing during reading (4; 5; 11; 14; 30). Furthermore, since word satiation is based on the perception of the word (45) and primarily relies on visual channels (15), this technology is the most effective in investigating visual information processing (51). Based on these features, it may be more advantageous for examining the phenomenon of word satiation in reading (46).

Therefore, this study adopted eye-tracking technology and designed two experiments to investigate the mechanism of word satiation in Tibetan reading. Experiment 1 manipulated two variables—repetition level and semantic relatedness—to investigate whether the word satiation originates from the loss of words’ semantics (i.e., semantic satiation). Experiment 2 manipulated two variables—repetition level and orthographic similarity—to investigate whether word satiation results from the loss of words’ morphology at the perceptual level (i.e., perceptual satiation). Previous studies had found that a priming effect occurred at a low repetition level; in contrast, no priming effect emerged or even reversed at a high repetition level. Due to the prolonged reaction time and decreased accuracy, satiation effects were triggered at the time (42). Researchers discovered that in category matching tasks, the reaction time in the semantically matching condition (e.g., fruit-apple) was longer than in the semantically mismatching one (e.g., fruit-ant), and that there was a semantic satiation effect (38; 39). Therefore, the following hypotheses were proposed in this study: (1) at a low repetition level, if fixation duration or reaction time are significantly shorter in the semantically related or orthographically similar conditions than in the semantically unrelated or orthographically dissimilar conditions, there will be semantic or orthographic priming effects; (2) at a higher repetition level, if fixation duration or reaction time is significantly longer in the semantically relevant or the orthographically similar conditions than in the semantically unrelated or orthographically dissimilar ones, there will be semantic or orthographic satiation effects.

## Experiment 1: Eye movement-based research of semantic satiation in Tibetan reading

This experiment used the eye-movement recording method combined with a category decision task for participants to determine whether the priming word and the target word were words of the same category, a task decision process that involves the processing of semantic information about the words. Two variables, repetition level and semantic relatedness, were manipulated to examine whether word satiation in Tibetan reading originated from a loss of semantic information about the words, i.e., whether it was semantic satiation.

### Participants

A total of 72 Tibetan university undergraduates who were native Tibetan speakers (37 males, *M-age* = 20.99) were recruited, with a Tibetan average score of 132.29 on the university entrance examination. They were all righthanded and had normal or corrected-to-normal vision, and no visual problems, including astigmatism and strabismus. Before the experiment, informed consent was obtained from all participants. After the experiment, all of them received 30 yuan as a reward.

### Design

A 2 (semantic relatedness: related, unrelated) × 3 (repetition level: low, medium, high) within-subject experimental design was adopted. The priming word was repeated 2-4 times at a low repetition level, 12-14 times at a medium repetition level, and 22-24 times at a high repetition level.

### Materials

Selection of experimental materials. Referring to Tian and Huber (42), 210 English common words were selected and translated into Tibetan. All words were 2.03 characters long on average and were divided into 70 groups, each group including a priming word and two target words (semantically related or unrelated to the priming words). There were respectively 70 semantically related and unrelated word pairs. Samples of the experimental materials are shown in Table 1.

**Table 1.** Samples of experimental materials.

Priming word	Semantically related target word	Semantically unrelated target word
ཞིང་པ།	འ5ོག་པ།	ཉི་མ།
Farmer	Herder	Sun

Evaluation of experimental materials. On a 5-point scale, we asked 20 homogeneous participants who did not take part in the formal study to score the familiarity of 210 words, the semantic relatedness of 140-word pairs (half semantically related, half semantically unrelated), and the orthographic similarity of 140-word pairs. Finally, 60 groups of words (practice materials: 6 groups; formal experimental materials: 54 groups) were selected as experimental materials. The evaluation results are shown in Table 2. Furthermore, t-test of semantic relatedness between semantically related and semantically unrelated word pairs was *t_118_* = 31.82, *p* < 0.001. The evaluation results revealed that the experimental materials were simple, the orthography of all word pairs was not similar, the semantic relatedness of semantically related word pairs was high, and the semantic relatedness of semantically unrelated word pairs was low. Thus, these materials were appropriate for our experimental requirements.

**Table 2.** The evaluation results of 60 groups of experimental materials.

Evaluation item	*M*	*SD*	Explanation
Familiarity	1.20	0.39	1 = very familiar, 5 = very unfamiliar
Semantic relevance (semantically related word pairs)	1.86	0.95	1 = very semantic-related, 5 = very semantically unrelated
Semantic relevance (semantically unrelated word pairs)	3.94	1.20	1 = very semantic-related, 5 = very semantically unrelated
Orthographic similarity	3.83	1.19	1 = very similar, 5 = very dissimilar

Arrangement of experimental materials. Each trial included six levels, which were divided into six blocks of 60 trials each. Each participant read one block, and after each trial, they assessed the semantic relatedness of the priming word and the target word. Therefore, each participant read a total of 60 trials.

### Apparatus

The SR Research Eyelink 1000 Plus eye tracker (sampling rate = 1000 Hz) was used to record eye movements. The materials were shown on a 24.5-inch DELL monitor (240 Hz sampling rate; 1920 x 1080 pixels resolution). The distance between the participants' eyes and the screen was approximately 65 centimeters. Microsoft Himalaya 36 typeface was used to show the information.

### Procedure

**Figure 1.**
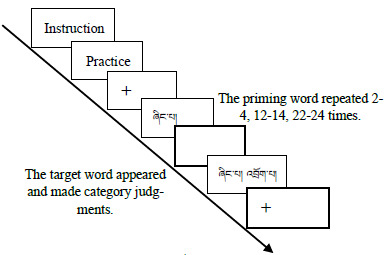
Experimental flow chart.

Each participant was tested individually. After entering the laboratory, participants were instructed to familiarize themselves with the surroundings before taking their assigned seat. The researcher then simply introduced the experimental procedure. Prior to the experiment, viewing positions were calibrated with a 3-point grid (error 0.25°) to ensure that the eye tracker could accurately record the participants’ eye movement trajectory (1; 13). Instructions were displayed on the test machine's screen after a successful calibration. The researcher next explained the requirements of the experiment to the participants. The experiment took about 20 minutes. The procedure (a single experimental trial) is shown in Figure 1.

## Results

In line with previous research (19; 18; 41; 44; 50), the analyzed indicators, including three eye movement measures and the reaction time, are as follows: (1) first fixation duration (FFD) refers to the duration of the first fixation on an area during first pass reading; (2) gaze duration (GD) refers to the sum of all fixations on an area from first entering the area until leaving it during first pass reading; (3) total fixation duration (TFD) refers to the sum of all fixations on a region; (4) reaction time (RT) refers to the time between a presentation (simultaneous presentation of the priming word and the target word) and a response. The FFD and GD represent the early stage of lexical processing, while the TFD represents the late stage (26; 52).

Each trial was divided into two areas of interest, with the priming word being the first and the target word being the second. The data on the target words were analyzed using the linear mixed model (LMM) and the lme4 package in the R environment (34; 2). The model enhanced the data utilization rate by incorporating all the original data and improved the reliability of the results by using the maximum random effect structure and integrating the participant and item effects. All indicators were log-transformed, and the regression coefficient (*b*), standard error (*SE*) and t value (*t* = *b/SE*) are reported in the results. If *|t|* > 1.96, it means *p* < 0.05.

Six participants were excluded (the accuracy rate was less than 85%), and the average accuracy rate for the remaining participants was 93%. To filter data, the following exclusion criteria were used (36; 37): (1) participants pressed the key prematurely or incorrectly during the experiment, which resulted in an interruption; (2) invalid data because of loss of tracking; (3) the single fixation duration was shorter than 80ms or longer than 1200ms. In total, 16% of the data were removed before conducting the analysis. The means and standard errors of indicators under all conditions are shown in Table 3. The results of statistical analysis are shown in Table 4.

**Table 3.** Means and standard errors of indicators under various conditions.

DV	Semantic status	Repetition level		
		Low	Medium	High
FFD	Semantically related	244 (5.64)	236 (6.11)	244 (6.96)
	Semantically unrelated	249 (6.39)	236 (7.22)	235 (6.50)
GD	Semantically related	710 (29.10)	716 (31.19)	709 (31.11)
	Semantically unrelated	776 (31.94)	719 (32.97)	673 (31.29)
TFD	Semantically related	874 (35.73)	955 (39.87)	1010 (44.47)
	Semantically unrelated	1001 (44.47)	954 (40.02)	939 (40.06)
RT	Semantically related	2048 (56.02)	2119 (59.37)	2222 (70.27)
	Semantically unrelated	2107 (75.05)	2046 (66.09)	2088 (68.91)

Note. DV is the dependent variable, the unit of each measure is millisecond, the values in parentheses are standard errors, The same as below..

**Table 4.**
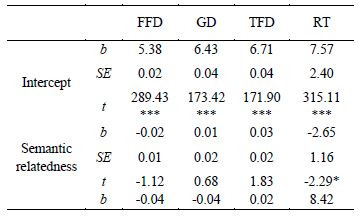

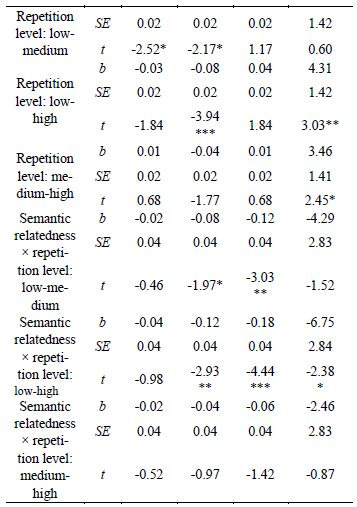
The results of statistical analysis of all indicators.

As shown in Table 4, the main effect of semantic relatedness was significant in RT, and the RT was significantly longer in the semantically related condition than in the semantically unrelated condition (*b* = 2.65, *SE* = 1.16, *t* = 2.29). Furthermore, the main effect of the repetition level was significant in the FFD, GD and RT (|*t*|*s* > 1.96, *ps* < 0.05). The FFD and GD were significantly longer at a low repetition level than at medium and high repetition levels, and the RT was significantly longer at a high repetition level than at low and medium repetition levels. Additionally, significant interaction between semantic relatedness and repetition level emerged in GD, TFD and RT (|*t*|*s* > 1.96, *ps* < 0.05). Further analysis found that at a low repetition level, there were significantly longer GD (*b* = 7.06, *SE* = 2.77, *t* = 2.55) and TFD (*b* = 1.22, *SE* = 2.73, *t* = 4.48) in the semantically unrelated condition than in the semantically related one; at a medium repetition level, the RT in the semantically related condition was significantly longer than that in the semantically unrelated one (*b* = 4.03, *SE* = 1.89, *t* = 2.13); at a high repetition level, there were significantly longer TFD (*b* = 5.42, *SE* = 2.76, *t* = 1.97) and RT (*b* = 6.15, *SE* = 1.91, *t* = 3.23) in the semantically related condition than in the semantically unrelated condition.

In summary, the interaction between semantic relatedness and repetition level differed significantly in terms of GD, TFD, and RT. There was a semantic priming effect in GD and TFD at a low repetition level (2 to 4 times), semantic satiation effects in RT at a medium repetition level (12 to 14 times) and semantic satiation effects in TFD and RT at a high repetition level (22 to 24 times). This finding revealed that there were effects of semantic priming and semantic satiation during lexical processing in Tibetan reading; furthermore, the satiation effect occurred in the late stage.

## Experiment 2: Eye movement-based research of perceptual word satiation in Tibetan reading

An orthographic similarity decision task with eyemovement recording method was used in this experiment, in which participants were asked to determine the orthographic similarity between the priming and target words. It was explored if word satiation in Tibetan reading is caused by a loss of word perceptual morphological information, or whether it is perceptual satiation, by controlling two variables: repetition level and orthographic similarity.

### Participants

Same as in Experiment 1.

### Design

A 2 (orthographic similarity: similar, dissimilar) × 3 (repetition level: low, medium, high) within-subject experimental design was used. The repetition levels were consistent with those in Experiment 1.

### Materials

Selection of experimental materials. We selected 210 common words from daily Tibetan expressions, with an average word length of 2.03 characters. The 210 words were divided into 70 groups. Each group included a priming word and two target words (similar or dissimilar orthography to the priming word). Examples of experimental materials are shown in Table 5.

Evaluation of experimental materials. Similarly, we invited 20 homogenous participants who did not participate in the formal experiment to rate the familiarity of 210 words, the orthographic similarity of 140 word pairs (half with similar orthography, half with different orthography), and the semantic relatedness of 140 word pairs on a 5-point scale. Finally, 60 groups of words were employed as experimental materials (practice materials: 6 groups, formal experimental materials: 54 groups). The evaluation results are shown in Table 6. Additionally, t-test of semantic relatedness between semantically related and semantically unrelated word pairs was *t_118_* = 49.52, *p* < 0.001, which was significant. The evaluation results showed that the experimental materials were simple, and that the semantics of all word pairs were irrelevant; word pairings of comparable orthography had high similarity, whereas word pairs with diverse orthography had low similarity. These experimental materials were appropriate for our requirements. The arrangement of experimental materials is identical to Experiment 1.

**Table 5.** Examples of experimental materials.

Priming word	Similar target word	Dissimilar target word
ཀོང་པ།ོ	ཀོང་ཇོ།	ཕོར་བ།
Lin Zhi	Princess	Leaves

**Table 6.** The evaluation result of 60 groups of experimental materials.

Evaluation item	*M*	*SD*	Explanation
Familiarity	1.16	0.49	1 = very familiar, 5 = very unfamiliar
Orthographic similarity (Similar pairs)	2.54	1.15	1 = very similar, 5 = very dissimilar
Orthographic similarity (dissimilar pairs)	4.51	0.88	1 =very similar, 5 = very dissimilar
Semantic relevance	4.24	1.03	1 = very semantically related, 5 = very semantically unrelated

### Apparatus and Procedure.

Same as in Experiment 1.

## Results

Six participants were excluded (4 participants’ accuracy rate was less than 70%, and two participants dropped out halfway), and the average accuracy rate of the remaining participants was 92%. The data deletion standard was identical to Experiment 1, and the deleted data accounted for approximately 13% of the total data. The analysis method was the same as in Experiment 1. Means and standard errors of all indicators in different conditions are shown in Table 7. The results of statistical analysis are shown in Table 8.

**Table 7.** Means and standard errors of all indicators under various conditions.

DV	Orthographic similarity	Repetition level		
		Low	Medium	High
FFD	Similar orthography	253 (5.87)	249 (6.62)	255 (7.78)
	Dissimilar orthography	286 (8.12)	273 (9.04)	271 (9.88)
GD	Similar orthography	496 (28.07)	531 (36.26)	522 (29.65)
	Dissimilar orthography	551 (21.56)	572 (25.99)	552 (24.51)
TRD	Similar orthography	656 (33.94)	713 (40.93)	736 (37.07)
	Dissimilar orthography	670 (31.62)	714 (33.08)	701 (33.31)
RT	Similar orthography	1747 (64.44)	1785 (67.61)	1821 (61.62)
	Dissimilar orthography	1483 (71.55)	1483 (57.25)	1477 (69.56)

**Table 8.**
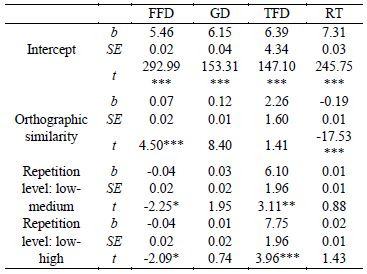

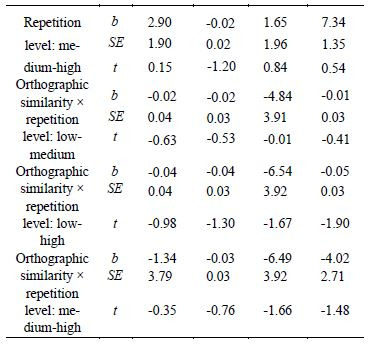
The results of statistical analysis of all indicators.

The results indicate that the main effect of orthographic similarity was significant in FFD (*b* = 0.07, *SE* = 0.02, *t* = 4.50) and RT (*b* = 0.19, *SE* = 0.01, *t* = 17.53). The FFD was significantly longer in the orthographic dissimilar condition than in the orthographic similar one, and the RT was significantly longer in the orthographic similar condition than in the orthographic dissimilar one. The main effect of the repetition level was significant in FFD and TFD (|*t*|s > 1.96, *p*s < 0.05), as well. The FFD was significantly longer at a low repetition level than at medium and high repetition levels. The TFD at medium and high repetition levels was significantly longer than at a low repetition level. There was no significant interaction between orthographic similarity and repetition level on all indicators (|*t*|s < 1.96, *p*s > 0.05).

Overall, the results demonstrated that in Tibetan reading, there was no orthographic priming or orthographic satiation effect during lexical processing.

## Discussion

### The mechanism of word satiation in Tibetan reading

This study has observed a semantic priming effect in Tibetan reading at low repetition levels, but semantic satiation effects at high repetition levels. The reasons for this result may be the following: (1) according to the semantic network activation model, in semantic memory, concepts are represented in the form of nodes which interconnect to form a semantic network. Therefore, when one conceptual node is activated, other interconnected nodes are also activated (8). When priming words are presented, their conceptual nodes are activated, and then target words’ (semantically related to the priming words) semantics are also activated. Therefore, the activation will further be strengthened at a low repetition level, leading to semantic priming effects. However, if the priming words are repeated frequently over a short period of time, the internal semantic representation will be heavily reactivated, resulting in fatigue in the semantic representation of the priming words. Not only will it be transmitted to the connected concept nodes but will also inhibit the semantic extraction of words which are semantically related to the priming word (29). The semantic priming effects will be decreased or perhaps reversed at this time, resulting in the emergence of the semantic satiation effect (38); (2) furthermore, the semantic satiation effect is not merely a reversal of the semantic priming effect, but may also be explained using cognitive neuroscience. It was stated that, if a stimulus is repeated over a short amount of time, the nervous system will be activated for a longer time. The constant activation will cause synaptic connections to be inhibited, resulting in a temporary loss of communication between transmitting and receiving neurons (17; 42; 43). The nervous system will be exhausted after repeatedly responding to priming words. This tiredness contributes to prolonged RT and GD on target words when they are presented. Following this, there is a semantic satiation effect. The results of this study are consistent in English reading, but not in Chinese reading (3; 21; 22; 23). Semantic satiation effects are the most common finding in English reading studies. The reason for this is that there is a precise orthography-to-phonology correspondence when reading alphabetic writing like English. The orthography will activate the phonology, and both the orthography and the phonology will access the semantics concurrently so that the word satiation in English reading tends to be semantic satiation. Tibetan and English, both using alphabetic writing, may demonstrate the commonality of the word satiation process. Inconsistently, orthographic satiation is the principal result of Chinese reading research (6; 7). The reason is that Chinese characters are logographic writing that only represents morphemes, not syllables (48), and the Chinese character is a hierarchical structure system built by strokes and components (24). Therefore, there is no consistent orthography-to-phonology mapping in Chinese. Furthermore, Chinese morphemes and radicals are densely packed with semantic information (31).

Consequently, readers are more reliant on “the route of orthography-to-semantics,” since it is simple to “see the orthography and know the semantics” but harder to “see the orthography and know the phonology” (20). Hence, the processing of Chinese characters relies more on orthogra-phy, and orthographic satiation is more likely to occur.

### The stage of semantic satiation effects in Tibetan reading

Studies have found that the semantic satiation effect in Tibetan reading was mainly significant on TFD. The TFD was sensitive to slower and longer cognitive pro-cessing (16), reflecting the late pro-cessing stage. Therefore, the semantic satiation effect in Tibetan reading mainly occurs in the late stage of lexical processing. The reasons may be as follows: first, the mate-rials (words) are highly familiar and commonly used in the daily life of native Tibetans. Readers need less cognitive resources when processing these words and, consequently, it takes a long time for them to reach satiation. Accord-ingly, the semantic satiation effect is difficult to occur in the early stage of lexical processing, and only exists in the relatively later stage of processing. Second, according to cognitive load theory, when cognitive resources are lim-ited, satiation operations (repetition of priming words) in-crease participants’ task and cognition loads, resulting in cognitive resource competition and attentive distribution problems, which are reflected in reduced judgment task ef-ficiency and the cost of response delay (40). Tong (44) also pointed out that the phenomenon of word satiation is inextricably connected to attention whose re-duction would be delayed with trigger satiation. In this study, the distributed attention resources reduced after the high repetition of the priming word. When the target words semantically related to the priming words were presented, readers needed to reactivate the semantics without quick response, resulting in the delay of semantic satiation. Therefore, the semantic satiation was triggered in the late stage of lexical processing.

### Conclusion

Word satiation in Tibetan reading does not emerge at the perceptual level of orthographic satiation but is semantic satiation. Moreover, semantic satiation is triggered in the late stage of lexical processing.

The findings of this study are compatible with those for English, but not with those for Chinese. The following three recommendations are based on these findings. First, researchers can further explore whether the word satiation varies from language to language. Second, people can utilize the word satiation mechanism to avoid linguistic recognition and writing faults, hence boosting reading and writing efficiency. Finally, we recommend that teachers should improve their teaching tactics based on language satiation principles. Teachers typically penalize pupils for repeatedly copying words many times. From the perspective of satiation, this strategy is very time-consuming for students and slows down their learning efficiency. Therefore, they can ask students to copy words 2 to 4 times at a low repetition level, leading to better teaching outcomes.

### Ethics and Conflict of Interest

All procedures performed in studies involving human participants were in accordance with the ethical standards of the institutional and national research committee and with the 1964 Helsinki Declaration and its later amendments or comparable ethical standards. The study was approved by Ethics Committee of Psychology of Tibet Autonomous Region. The authors have no competing interests to declare that are relevant to the content of this article.

### Acknowledgements

This work was supported by grants from the National Natural Science Foundation of China (31860280, 32260204).

We thank all authors who contributed to the conception and design of this study. We appreciate Shan Li, Zibei Niu, and Danhui Wang’s help with the preparation of the study’s materials, data collection, and analysis. Additionally, we are very grateful Xiuling Li and Zeng Man for drafting the manuscript. We sincerely appreciate the assistance in revising the manuscript that Ms. Lei Gao, Mr. Tianzhi Li, Mr. Xuejun Bai, and Mr. Xiaolei Gao provided. The funding and resources provided by Mr. Xiaolei Gao are greatly appreciated.
